# Miscellaneous

**Published:** 1888-09

**Authors:** 


### MISCELLANEOUS.

Dr. Holmes' Carpenter Shop.—A favorite corner with Dr. Holmes in the Beacon street home, says the *Book Buyer*, is in the lower basement. Here opposite shelves of books, is fitted up a small carpenter shop, to which the poet often resorts. Chisels, hammers, nails and files are all in their proper places, while before the window stands a foot-lathe. "It is pleasant exercise to tread this, and see it do its work so quickly and neatly," says Dr. Holmes, with a merry twinkle in his eye, as he picks up a small piece of wood and watches the borer's work as it twirls through it. And here, almost daily, is found the genial poet at the carpenter's bench, doing some odd little job as a diversion from singing and writing his way into the hearts of thousands who hope it may be a long time before

"By life's decaying fire
His fingers sweep the stringless lyre."

The Theory of Massage.—*Medical Register* of Feb. 25th, gives a novel theory as to the action of massage. It says: We know massage is beneficial as we know the tongue to be coated in certain fevers, but why this occurs may puzzle many to whom the mysteries of physiology are too tightly sealed· In a word, then the theory of massage is based not only upon the known effects already referred to, but upon the hypothesis of the direct alteration in the structure of cells. In disease the cell walls in the animal tissue become hypertrophied. The formative material or nucleus is imprisoned in an impenetrable sheath throngh which nourishment cannot be carried. This hypertrophy of the cell wall in pathological conditions is identical with that which results by a slow process from the advancement of age. Massage breaks down this hypertrophied condition of the cell wall by mechanical means, creating fissures through which nourishment forces its way to the nucleus within. Theoretically, if massage could be applied to every cell of the body, there would be no reason why we should ever grow old ; but, this being practically impossible, we must be content with the more limited benefits it bestows, which, however, are important and considerable."

Liquid Iron-Rio.—Product of the Rio Chemical Co., St. Louis. This is the best preparation of Iron for medicinal purposes of which we have any knowledge. The Solution is so complete and thoroughly held that there is no tendency in the iron to settle and form a sediment as in many similar compounds, besides being agreeable to the stomach ; which ensures its retention. Such is the unamimous opinion after tests of our entire medical staff.

The Medical Fraternity of Brazil appears to be of a mixed nationality. There are many natives practising there who were educated in France and Germany; the French and Portuguese are, however, in the majority.

There is a remarable and self-imposed family law in relation to matrimony which prevails, we believe, throughout Brazil. It is recognized among all the higher classes. The man who is about to marry is required to furnish a certificate from one or more physicians that he is free from diseases of a certain character, and that he is free, also, from all signs of any of the diseases which are liable to be transmitted to the offspring. Not only that, but the physicians consulted must testify, that as far as they can learn, there exists no reason to believe that the union will be other than in accord with the laws of sanitation. DR. ARONY.

SICK HEADACHE.—The periodical recurrence of sick headache with many persons, is a grievous affliction. Those who suffer from it should correct every habit and avoid all indiscretions which they know are likely to be followed by an attack. They should also overcome every derangement of the system which exists, if possible, end strengthen every part and function of the same. In fact, they should treat at first not the head and its aches, but endeavor to build up the general health. In the attempt to do so they must not indiscriminately dose themselves with drugs, but rather depend upon pure air, exercise, sufficient sleep, good wholesome food, and other measures of like character.

One of the greatest essentials in treatment will be a careful selection of the diet, and a rigid restriction to those articles of food, which, in their experience, has proved the least burdensome to their digestive organs. There is no dietary which is alike suited to all. Each must learn what, and how much, is the proper for him or her to eat, and what should be avoided ; and those substances which are known to be difficult of digestion should never be indulged in. While careful not to tax the stomach they must keep the bowels active. If constipation exists, headaches are quite certain to occur.

Under the simple treatment advised, if properly employed and persisted in, many who are victims of sick headaches will suffer much less often, or escape entirely, those distressing visitations.

### COMPENSATION.

BY HORACE M. RICHARDS.

If you lift from some heart its burden of care,
As you journey o'er life's dusty road,
You not only are garnering treasures up there,
But you lighten your own earthly load.

If you cheer some soul on its wearisome way,
Or drive from some brow its shadows and gloom,
Your burden will lighten each hour of the day,
And you are strewing with flowers your road to the tomb.

If you come as a helper to a soul that's in need,
Or lend to the weary your strengthening hand,
You are tilling God's garden and sowing the seed
For a harvest of love, in the soul's summer land.

If you have but a word, a smile, or a tear,
Don't hoard it, give freely, 'twill solace some grief,
Take the pain from some heart, some weary one cheer
And bring to the pain in thine own heart, relief.

